# Effects of single and combined applications of entomopathogenic fungi and nematodes against *Rhynchophorus ferrugineus* (Olivier)

**DOI:** 10.1038/s41598-017-05615-3

**Published:** 2017-07-20

**Authors:** Waqas Wakil, Muhammad Yasin, David Shapiro-Ilan

**Affiliations:** 1 Department of Entomology, University of Agriculture, Faisalabad, Pakistan; 2 College of Agriculture, Bahauddin Zakariya University Bahadur Sub-Campus, Layyah, Pakistan; 3 USDA-ARS, SEFTNRL, Byron, GA USA

## Abstract

This study was carried out to investigate the insecticidal properties of *Beauveria bassiana*, *Metarhizium anisopliae* and *Heterorhabditis bacteriophora* for their virulence against different larval instars of *Rhynchophorus ferrugineus* (Olivier). Both fungi were either applied alone or in combination, with *H. bacteriophora* simultaneously or 1 and 2 weeks after fungal application; EPN were also applied alone. Moreover, assessment of host development, diet consumption, frass production and weight gain were observed at sub-lethal dose rates. In combined treatments, additive and synergistic interactions were observed. Synergism was observed more frequently in *H. bacteriophora* + *B. bassiana* combinations than in *H. bacteriophora* + *M. anisopliae* combinations, and was higher in early instars than old instars. In 2^nd^ and 4^th^ instars, synergy was noted in *H. bacteriophora* + *B. bassiana* combinations at 0, 7 and 14 d intervals and in 6^th^ instar synergy was observed only in *H. bacteriophora* + *B. bassiana* combinations (at 0 and 7 d intervals). A decrease in pupation, adult emergence and egg hatching was enhanced in the combined treatments. Furthermore, reduced weights and variation in duration of insect developmental stages were observed among entomopathogens and enhanced in *H. bacteriophora* + *B. bassiana* combinations. Larvae treated with sub-lethal concentrations exhibited reductions in food consumption, growth and frass production and weight gain.

## Introduction

The coleopteran insect pests are ranked among the most voracious pests of economically important crops. Among these notorious insect pests, red palm weevil (RPW) *Rhynchophorus ferrugineus* (Olivier) (Coleoptera: Curculionidae) is highly destructive; the insect devastates 29 different palm species, particularly date palms which are economically important crops in the Middle East, Africa and South East Asia^1, 2^. Synonymously, the pest is known as the Asiatic palm weevil, coconut weevil, red stripe weevil, and hidden enemy, and also called AIDS of palm because of the damage caused and the resulting slow death of palm trees^3^. The pest has a cryptic nature and mostly damages palm trees younger than 20 years^4, 5^ for which the crown, trunk and bole are the natural sites of damage. The crowns are the sites of infestation in older plantations. The larvae spend their early stages within the tree trunk, destroying the vascular system and boring into the heart of host, which may lead to tree collapse^6^. The neonate larvae chew plant fibers and advance towards the interior leaving behind the chewed-up frass which has a typical fermented odor. The completely developed grubs pupate in a cocoon fabricated from chewed fibers, and pupal period lasts for 11–45 days. Adult weevil can interbreed and live within the same host until they are required to colonize to a new palm. If the plant remains untreated the palm can die within 6–8 months^7–9^.

To combat this insect pest, synthetic insecticides and fumigants have remained the mainstay of date palm growers for decades. However, chemical control is challenging due to the cryptic nature of RPW^10^. Moreover, the chemical insecticides have exerted negative effects on the environment and human health, and the pest has developed resistance against many of these chemicals^5^. Alternatively, entomopathogens may have potential for control of *R. ferrugineus*. Among microbial control agents, entomopathogenic fungi (EPFs) particularly *Beauveria bassiana* s.l. (Ascomycota: Hypocreales) and *Metarhizium anisopliae* s.l. (Ascomycota: Hypocreales) are considered promising alternatives to conventional synthetic insecticides. They pose negligible detrimental effect on environment and human health^11^, and harbor promising insecticidal activities against a number of arthropod pests^12, 13^.

Several researchers have isolated and successfully deployed these two fungal species against different developmental stages of RPW as biocontrol agents both under laboratory and field conditions^14–26^. EPFs are attractive relative to a number of other microbial agents due to their novel mode of action by direct contact to the host cuticle instead of ingestion, and their ability to transfer inoculum from treated insects to untreated insects via the new generation of spores^27^.

Similarly, entomopathogenic nematodes (EPNs) are also promising microbial control agents and are efficient in suppressing a variety of insect pests^28–32^. They are obligate parasites in the families Steinernematidae and Heterorhabditidae which kill insects with the aid of mutualistic bacterium carried in their intestine (*Xenorhabdus* spp. and *Photorhabdus* spp. bacteria are associated with *Steinernema* spp. and *Heterorhabditis* spp., respectively)^33^. Both microbial agents (fungi and nematodes) are considered safe to non-target organisms (vertebrates and invertebrates) and the environment, and they can be successfully integrated with each other, often exhibiting strong additive and synergistic interactions^34–37^. A number of scientists have evaluated different EPN species against RPW both under laboratory and field conditions^28, 29, 38–47^.

The objective of this study was to explore the potential for integration of *B. bassiana, M. anisopliae* and *H. bacteriophora* in a control program for *R. ferrugineus*. Mortality, development and growth of *R. ferrugineus* was studied under laboratory conditions following application of fungus and nematode treatments applied alone, or fungus-nematode combinations. Interactions (synergy, additivity or antagonism) between microbial agents were assessed. Results will assist in decision-making for selecting the most suitable treatments and application times of both agents in future field trials, and eventually lead to successful and environment friendly control of *R. ferrugineus* populations in date palm systems.

## Results

### Entomopathogenic fungi and nematodes interaction

In integrated application of *H. bacteriophora* with *B. bassiana* or *M. anisopliae*, additive and synergistic interactions were observed in all the three instars tested when the microbial agents were applied simultaneously or following delayed nematode application (Tables 1, 2 and 3). Antagonism was not observed in any of the combinations tested (Tables 1, 2 and 3). During simultaneous application, 2^nd^ instar larvae exhibited additive effects for *B. bassiana* and *H. bacteriophora* for the first two weeks, while synergistic interaction was observed at the third week of application. The degree of synergism increased with the delayed application of *H. bacteriophora* one or two weeks after *B. bassiana* treatments. For *M. anisopliae* additive effects were recorded for simultaneous application, while interactions were shifted towards synergism when delayed nematode application was made after one and two weeks of fungal spore application (Table 1). A similar trend was recorded for 4^th^ and 6^th^ instar larvae but susceptibility was lower among treatments as compared with the 2^nd^ instar larvae, and the instances of synergy also decreased as instar size increased (Tables 1, 2 and 3). Also, synergy was observed in *H. bacteriophora* and *B. bassiana* combinations more than in *H. bacteriophora* and *M. anisopliae* combinations (Tables 1, 2 and 3).

**Table 1:** Mean mortality (% ± SE) of 2^nd^ instar larvae of *Rhynchophorus ferrugineus* treated with *Beauveria bassiana, Metarhizium anisopliae* and *Heterorhabditis bacteriophora. B. bassiana* and *M. anisopliae* were used each @ 1 × 10^6^ conidia ml^−1^ and *H. bacteriophora* was applied @ 100 IJs ml^−1^ (Bb: *Beauveria bassiana*, Ma: *Metarhizium anisopliae*, EPN: *Heterorhabditis bacteriophora*, IJs: Infective juveniles).

Treatments	Intervals^a^	Week^b^	Observed mortality	Expected mortality	Chi Sq.	Type of interaction
Bb	—	1	11.22	—	—	—
	—	2	14.28	—	—	—
	—	3	20.40	—	—	—
Ma	—	1	8.16	—	—	—
	—	2	12.24	—	—	—
	—	3	17.34	—	—	—
EPN	—	1	14.28	—	—	—
	—	2	21.42	—	—	—
	—	3	29.59	—	—	—
Bb + EPN	0	1	27.55	23.90	0.48	Additive
	0	2	43.87	32.65	2.87	Additive
	0	3	61.22	43.96	4.86	Synergistic
Ma + EPN	0	1	23.71	21.28	0.24	Additive
	0	2	32.98	38.21	0.82	Additive
	0	3	48.45	36.94	2.73	Additive
Bb + EPN	7	1	32.99	26.53	1.26	Additive
	7	2	51.54	37.46	3.84	Synergistic
	7	3	73.19	51.86	6.21	Synergistic
Ma + EPN	7	1	28.86	24.78	0.57	Additive
	7	2	45.36	35.05	2.33	Additive
	7	3	64.94	48.27	4.28	Synergistic
Bb + EPN	14	1	51.54	37.46	3.84	Synergistic
	14	2	69.07	51.86	4.28	Synergistic
	14	3	88.65	62.51	7.70	Synergistic
Ma + EPN	14	1	44.32	35.05	1.93	Additive
	14	2	64.94	48.99	3.92	Synergistic
	14	3	75.25	53.95	6.02	Synergistic

^a^Intervals between the application of EPFs and EPNs. ^b^Week after fungal application.

**Table 2:** Mean mortality (% ± SE) of 4^th^ instar larvae of *Rhynchophorus ferrugineus* treated with *Beauveria bassiana, Metarhizium anisopliae* and *Heterorhabditis bacteriophora. B. bassiana* and *M. anisopliae* were used each @ 1 × 10^6^ conidia ml^−1^ and *H. bacteriophora* was applied @ 100 IJs ml^−1^ (Bb: *Beauveria bassiana*, Ma: *Metarhizium anisopliae*, EPN: *Heterorhabditis bacteriophora*, IJs: Infective Juveniles).

Treatments	Intervals^a^	Week^b^	Observed mortality	Expected mortality	Chi Sq.	Type of interaction
Bb	—	1	9.18	—	—	—
	—	2	11.22	—	—	—
	—	3	16.32	—	—	—
Ma	—	1	6.12	—	—	—
	—	2	9.18	—	—	—
	—	3	14.28	—	—	—
EPN	—	1	12.24	—	—	—
	—	2	17.34	—	—	—
	—	3	23.46	—	—	—
Bb + EPN	0	1	24.29	20.30	0.71	Additive
	0	2	34.69	26.62	1.87	Additive
	0	3	51.02	35.96	4.44	Synergistic
Ma + EPN	0	1	19.58	17.61	0.19	Additive
	0	2	28.86	24.93	0.53	Additive
	0	3	44.32	34.40	2.22	Additive
Bb + EPN	7	1	27.83	22.09	1.18	Additive
	7	2	43.29	30.84	3.58	Additive
	7	3	61.85	42.99	5.75	Synergistic
Ma + EPN	7	1	23.71	20.30	0.48	Additive
	7	2	38.14	29.15	2.11	Additive
	7	3	55.67	40.64	4.05	Synergistic
Bb + EPN	14	1	42.26	30.84	3.08	Additive
	14	2	58.76	42.99	4.23	Synergistic
	14	3	80.41	57.39	6.58	Synergistic
Ma + EPN	14	1	36.08	29.15	1.33	Additive
	14	2	54.63	40.64	3.58	Additive
	14	3	72.16	53.95	4.50	Synergistic

^a^Intervals between the application of EPFs and EPNs. ^b^Week after fungal application.

**Table 3:** Mean mortality (% ± SE) of 6^th^ instar larvae of *Rhynchophorus ferrugineus* treated with *Beauveria bassiana, Metarhizium anisopliae* and *Heterorhabditis bacteriophora. B. bassiana* and *M. anisopliae* were used each @ 1 × 10^6^ conidia ml^−1^ and *H. Bacteriophora* was applied @ 100 IJs ml^−1^ (Bb: *Beauveria bassiana*, Ma: *Metarhizium anisopliae*, EPN: *Heterorhabditis bacteriophora*, IJs: Infective Juveniles).

Treatments	Intervals^a^	Week^b^	Observed mortality (%)	Expected mortality	Chi Sq.	Type of interaction
Bb	—	1	7.14	—	—	—
	—	2	9.18	—	—	—
	—	3	13.26	—	—	—
Ma	—	1	4.081	—	—	—
	—	2	7.14	—	—	—
	—	3	11.22	—	—	—
EPN	—	1	9.18	—	—	—
	—	2	14.28	—	—	—
	—	3	18.36	—	—	—
Bb + EPN	0	1	17.34	15.67	0.16	Additive
	0	2	28.57	22.15	1.43	Additive
	0	3	42.85	29.19	4.35	Synergistic
Ma + EPN	0	1	13.40	12.89	0.01	Additive
	0	2	22.68	20.40	0.22	Additive
	0	3	35.05	27.53	1.61	Additive
Bb + EPN	7	1	21.64	17.52	0.78	Additive
	7	2	35.05	25.65	2.51	Additive
	7	3	50.51	35.02	4.74	Synergistic
Ma + EPN	7	1	17.52	15.67	0.19	Additive
	7	2	30.92	23.90	1.59	Additive
	7	3	44.32	31.69	3.60	Additive
Bb + EPN	14	1	34.02	25.65	2.05	Additive
	14	2	48.45	35.02	3.72	Additive
	14	3	71.13	51.86	5.22	Additive
Ma + EPN	14	1	28.86	23.90	0.85	Additive
	14	2	42.26	31.69	2.64	Additive
	14	3	61.85	46.03	4.04	Additive

^a^Intervals between the application of EPFs and EPNs. ^b^Week after fungal application.

In a factorial analysis the main effects for pupation adult emergence and egg eclosion were significant while their interaction effects were non-significant except pupation (Table 4). A decrease in pupation, adult emergence and egg hatching was caused by all treatments and the effects were enhanced in the combined treatments (particularly with *H. bacteriophora* + *B. bassiana*) (Table 5).

**Table 4:** Factorial analysis for pupation, adult emergence and egg eclosion of *Rhynchophorus ferrugineus* exposed to *Beauveria bassiana, Metarhizium anisopliae* and *Heterorhabditis bacteriophora*.

Source	*df*	Pupation		Adult emergence		Egg eclosion	
		*F*	*P*	*F*	*P*	*F*	*P*
Instar	2	18.78	<0.01	39.94	<0.01	35.0	<0.01
Treatment	9	114.28	<0.01	84.44	<0.01	90.89	<0.01
Instar × Treatment	18	10.49	<0.01	0.48	0.96	0.46	0.97
Error	232	—	—	—	—	—	—
Total	269	—	—	—	—	—	—

**Table 5:** Pupation, adult emergence and egg eclosion (% ± SE) of 2^nd^, 4^th^ and 6^th^ instar *R. ferrugineus* larvae treated with *Beauveria bassiana* (1 × 10^6^ spore ml^−1^)*, Metarhizium anisopliae* (1 × 10^6^ spore ml^−1^) and *Heterorhabditis bacteriophora* (100 IJs ml^−1^). Mean sharing the same letters are not significantly different at 5% level (Bb: *Beauveria bassiana*, Ma: *Metarhizium anisopliae*, EPN: *Heterorhabditis bacteriophora*, IJs: Infective Juveniles).

Treatment	Interval	Second instar			Fourth instar			Sixth instar		
		Pupation (%)	Adult emergence (%)	Egg eclosion (%)	Pupation (%)	Adult emergence (%)	Egg eclosion (%)	Pupation (%)	Adult emergence (%)	Egg eclosion (%)
Bb	—	62.22 ± 2.23bc	57.77 ± 2.77bc	53.33 ± 2.33bc	71.11 ± 3.54b	66.66 ± 3.08b	59.55 ± 2.92b	80.00 ± 2.88bc	74.44 ± 3.37b	66.33 ± 3.10b
Ma	—	67.77 ± 2.64b	61.11 ± 3.21b	58.88 ± 2.23b	73.33 ± 3.40b	68.88 ± 3.51b	62.22 ± 2.77b	83.33 ± 2.35ab	78.88 ± 3.88ab	72.22 ± 3.22b
EPN	—	56.66 ± 2.33bcd	50.55 ± 2.69bcd	45.55 ± 1.67bcd	62.22 ± 2.77bc	57.77 ± 2.33bc	51.11 ± 2.51bc	69.44 ± 3.42 cd	65.55 ± 2.75bc	59.33 ± 2.44bc
Bb + EPN	0	45.55 ± 1.93def	40.33 ± 2.35def	36.66 ± 1.88def	48.88 ± 2.09 cd	43.33 ± 3.08 cd	38.88 ± 1.60 cd	54.44 ± 2.76ef	49.44 ± 2.57de	43.33 ± 2.35de
Ma + EPN	0	51.11 ± 1.51cde	44.77 ± 2.89cde	39.44 ± 1.36cde	54.44 ± 2.12 cd	49.44 ± 2.16 cd	44.44 ± 2.23 cd	60.00 ± 2.88de	56.66 ± 2.72 cd	51.11 ± 2.51 cd
Bb + EPN	7	39.44 ± 1.69ef	36.66 ± 1.33def	31.11 ± 1.51def	45.55 ± 2.42d	40.55 ± 1.93d	35.55 ± 1.93d	51.11 ± 2.51ef	45.55 ± 2.93de	40.55 ± 1.42de
Ma + EPN	7	43.55 ± 1.73def	38.88 ± 1.51def	34.44 ± 1.37def	51.11 ± 2.88 cd	46.11 ± 2.32 cd	41.11 ± 1.51 cd	57.77 ± 2.77def	52.22 ± 2.23cde	46.66 ± 1.33cde
Bb + EPN	14	32.22 ± 1.27 f	27.77 ± 1.77 f	22.22 ± 1.46 f	39.44 ± 2.42d	36.66 ± 1.33d	31.11 ± 1.60d	44.44 ± 2.93 f	40.22 ± 2.79e	34.44 ± 1.93e
Ma + EPN	14	35.55 ± 1.43 f	30.55 ± 3.37ef	26.66 ± 1.68ef	47.77 ± 2.23 cd	42.22 ± 2.12 cd	37.77 ± 1.22 cd	54.44 ± 2.76ef	47.77 ± 2.64de	42.22 ± 1.22de
Control		90.55 ± 2.11a	86.66 ± 2.88a	81.11 ± 2.60a	93.33 ± 2.66a	90.55 ± 2.11a	83.33 ± 3.33a	95.55 ± 1.75a	92.22 ± 2.22a	85.55 ± 2.93a
*df*		9	9	9	9	9	9	9	9	9
*F*		35.7	31.3	31.0	23.4	23.6	28.8	30.6	28.1	32.4
*P*		<0.01	<0.01	<0.01	<0.01	<0.01	<0.01	<0.01	<0.01	<0.01

### Development of *R. ferrugineus*: larvae through adult stages

Growth and development of RPW (larvae to adult stage) was adversely affected by the microbial agents. When larvae were exposed to the sub-lethal doses of *H. bacteriophora*, *B. bassiana* and *M. anisopliae*, significant variations were recorded for larval duration, larval weight, pre-pupal duration, pre-pupal weight, pupal duration, pupal weight, adult longevity and adult weight (larval duration: *F*_5, 53_ = 9.92, P <0.01; larval weight *F*_5, 53_ = 27.3, P <0.01; pre-pupal duration: *F*_5, 53_ = 6.59, P <0.01; pre-pupal weight: *F*_5, 53_ = 6.94, P<0.01; pupal duration *F*_5, 53_ = 5.15, P < 0.01; pupal weight *F*_5, 53_ = 11.10, P <0.01; adult longevity (female *F*_5, 53_ = 3.93, P < 0.01 and male *F*_5, 53_ = 5.58, P < 0.01); adult weight (female *F*_5, 53_ = 4.26, P < 0.01 and male *F*_5, 53_ = 12.7, P < 0.01). For the most part, increases in larval, pre-pupal and pupal duration occurred while decreases in weight among stages were observed for all the treatments tested. On the other hand a decrease in adult life span and weight (male and female) was also recorded. The numerically highest detrimental effect on growth tended to be for combined application of *B. bassiana* and *H. bacteriophora* (e.g., see larval and adult weights) followed by *M. anisopliae* and *H. bacteriophora, H. bacteriophora* alone, *B. bassiana* and *M. anisopliae* (Table 6).

**Table 6:** Effect of *Beauveria bassiana* (1 × 10^4^ spore ml^−1^)*, Metarhizium anisopliae* (1 × 10^4^ spore ml^−1^) and *Heterorhabditis bacteriophora* (50 IJs ml^−1^) on the development of *Rhynchophorus ferrugineus*. Mean sharing the same letters within each column are not significantly different at 5% level (Bb: *Beauveria bassiana*, Ma: *Metarhizium anisopliae*, EPN: *Heterorhabditis bacteriophora*, IJs: Infective Juveniles).

Treatments	Larval duration (days)	Larval weight (g)	Pre-pupal duration (days)	Pre-pupal weight (g)	Pupal duration (days)	Pupal weight (g)	Adult longevity (days)		Adult weight (g)	
							Male	Female	Male	Female
Bb	98.16 ± 3.21b	4.01 ± 0.12bc	15.16 ± 0.58bc	4.02 ± 0.20ab	22.94 ± 1.20bc	3.92 ± 0.18abc	39.05 ± 1.54ab	42.83 ± 1.78ab	1.411 ± 0.12ab	1.14 ± 0.11abc
Ma	96.50 ± 3.88bc	4.41 ± 0.15ab	15.94 ± 0.79bc	4.07 ± 0.19ab	23.72 ± 1.36abc	4.11 ± 0.15ab	41.83 ± 1.40a	43.61 ± 1.67a	1.33 ± 0.14ab	1.28 ± 0.15ab
EPN	101.16 ± 3.63ab	3.72 ± 0.12 cd	16.72 ± 0.95abc	3.85 ± 0.14ab	24.16 ± 1.41abc	3.67 ± 0.10bcd	37.16 ± 1.23ab	40.38 ± 1.45ab	1.18 ± 0.1bc	1.05 ± 0.12abc
Bb + EPN	109.27 ± 4.16a	3.08 ± 0.12e	20.16 ± 1.14a	3.07 ± 0.11c	27.61 ± 1.24a	3.21 ± 0.11d	34.94 ± 1.29b	36.50 ± 1.78b	0.84 ± 0.10d	0.78 ± 0.11c
Ma + EPN	104.38 ± 3.32ab	3.27 ± 0.11de	18.50 ± 0.98ab	3.48 ± 0.12bc	25.50 ± 1.41ab	3.43 ± 0.13 cd	36.16 ± 1.17b	38.71 ± 1.68ab	1.01 ± 0.17 cd	0.92 ± 0.16bc
Control	87.05 ± 1.08c	4.87 ± 0.14a	14.38 ± 0.74c	4.17 ± 0.25a	21.27 ± 1.36c	4.24 ± 0.10a	42.83 ± 1.61a	45.27 ± 1.43a	1.56 ± 0.13a	1.37 ± 0.11a
*df*	5	5	5	5	5	5	5	5	5	5
*F*	9.92	27.3	6.57	6.94	5.18	11.1	5.55	3.92	12.7	4.26
*P*	<0.01	<0.01	<0.01	<0.01	<0.01	<0.01	<0.01	<0.01	<0.01	<0.01

### Development of *R. ferrugineus*: focus on last instar

**Figure 1:**
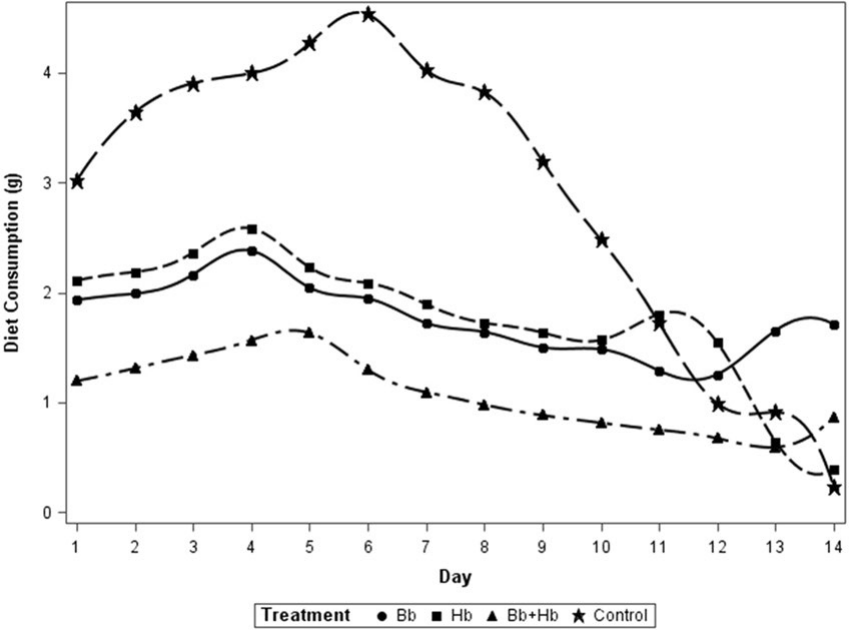
Diet consumption (g) in last instar larvae of *Rhynchophorus ferrugineus* when treated with *B. bassiana* (1 × 10^4^ spore ml^−1^) and *H. bacteriophora* (50 IJs ml^−1^); (Bb: *Beauveria bassiana*, Hb: *Heterorhabditis bacteriophora*, IJs: Infective Juveniles).

**Figure 2:**
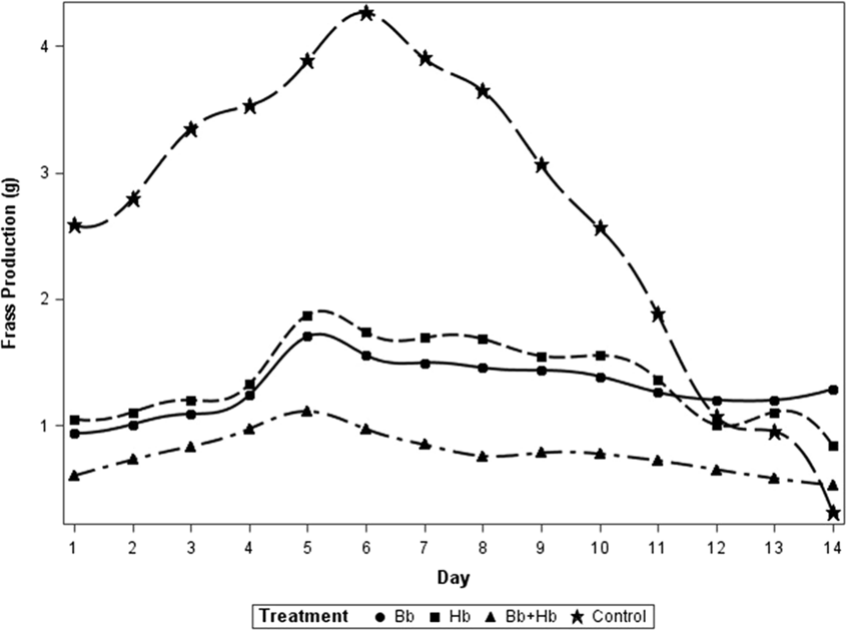
Frass production (g) in last instar larvae of *Rhynchophorus ferrugineus* when treated with *B. bassiana* (1 × 10^4^ spore ml^−1^) and *H. bacteriophora* (50 IJs ml^−1^); (Bb: *Beauveria bassiana*, Hb: *Heterorhabditis bacteriophora*, IJs: Infective Juveniles).

**Figure 3:**
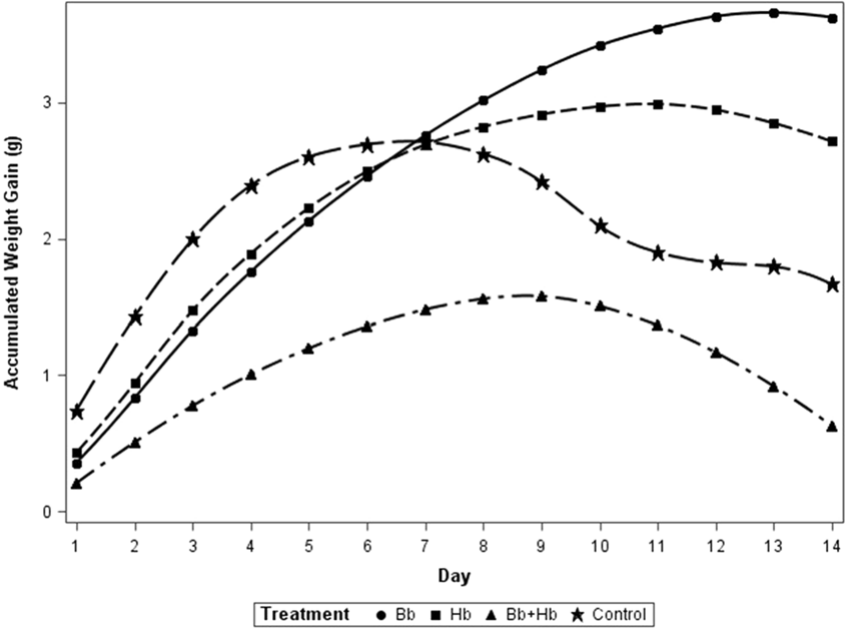
Cumulative weight gain (g) in last instar larvae of *Rhynchophorus ferrugineus* when treated with *B. bassiana* (1 × 10^4^ spore ml^−1^) and *H. bacteriophora* (50 IJs ml^−1^); (Bb: *Beauveria bassiana*, Hb: *Heterorhabditis bacteriophora*, IJs: Infective Juveniles).

Based on repeated measures analysis (and Tukey’s separation), diet consumption by 10^th^ instar was significantly influenced by the treatments applied (*F*_3, 63_ = 250.67, P < 0.01); diet consumption was lowest in the combined treatment of *H. bacteriophora* and *B. bassiana* compared to their sole applications and all treatments had lower consumption than the control (Fig. 1). Similarly frass production was influenced by treatments applied (*F*_3, 69_ = 386.22, P <0.01), with the lowest frass production for the combined treatment of *H. bacteriophora* and *B. bassiana* (0.57 ± 0.04 to 0 ± 0.00 g) and highest in the control (the single-applied treatments were intermediate and not different from each other) (Fig. 2). Untreated larvae (control) and larvae treated with sub-lethal concentrations of *B. bassiana* and *H. bacteriophora* gained more weight as compared to their combined application (*F*_3, 24_ = 56.77, P <0.01) (Fig. 3); the single applied treatments were not different from the control whereas the combination treatment was statistically separated from all three others.

## Discussion

This is the first study to investigate the combined effects of fungal isolates and *H. bacteriophora* against larvae of RPW. The results revealed that the microbial agents applied alone displayed pathogenicity. However, when fungi and nematodes were combined (either applied simultaneously or delayed application of *H. bacteriophora*), the level of virulence was enhanced in an additive or synergistic fashion. Synergistic levels of virulence were observed more frequently in *H. bacteriophora* + *B. bassiana* combinations than in *H. bacteriophora* and *M. anisopliae* combinations. Additionally, synergy appeared to be more prevalent in the younger instars tested than the later instars, and our findings are similar to the findings of Ansari *et al*.^48, 49^ indicating positive interactions with the combined application of *H. megidis* or *S. glaseri* with *M. anisopliae* CLO 53 against 3^rd^ instar *H. philanthus* under laboratory and greenhouse conditions, and between *H. bacteriophora* and *M. anisopliae* CLO 53 under field conditions. Similarly synergistic effects were observed in combined treatments of *H. bacteriophora* and *M. anisopliae* isolate against barley chafer grub, *C. curtipennis* after 5 weeks of *M. anisopliae* application^50^. They have suggested exposing grubs 3 or 4 weeks before addition of nematodes to get stronger synergistic interactions. In our study enhanced efficacy and stronger interactions were recorded at 1 or 2 weeks delayed application of *H. bacteriophora*.

In prior research, synergistic interactions were also observed between entomopathogenic nematodes and fungi. For example^51^ laboratory trials were conducted against *Otiorhynchus sulcatus* (the black vine weevil) using a combination of *M. anisopliae* and EPN. Synergy occurred with either *H. bacteriophora* or *S. kraussei* and mortality with *S. feltiae* was recorded as additive. They also performed greenhouse trials with the combination of these agents applied at various concentrations. The combination of *M. anisopliae* and *H. bacteriophora* resulted in synergistic interactions in all treatments. The combinations of *M. anisopliae* and *S. feltiae* only resulted in synergy when a high concentration of fungus was used with a low concentration of nematodes; all other interactions were additive^51^. When overwintering black vine weevils in greenhouses were exposed to *M. anisopliae* and *S. kraussei* the interaction recorded was synergistic in the first trial, but when the trial was repeated results were additive^52^. This shows that the combination of these pathogens may not be consistent. It was hypothesised that the inconsistent results may be due to the sensitivity of the fungus to temperature^52^.

We speculate that the longer grubs are exposed to the fungus, the more debilitated they become and subsequently are more susceptible to the EPN. It is also conceivable that the debilitated insects respire more, attracting the EPNs, which follow a CO_2_ gradient to their hosts^51^. It is also suggested that the stressed insects are more vulnerable to pathogen infection, which thus enhances insect mortality or facilitates a higher speed of kill leading to synergistic effects in combined treatments^53^. For example, *Paenibacillus popilliae* (Dutky) applied against scarab larvae acted as a stressor to nematode infection which caused elevated larval mortality^34, 35^. Similarly entomopathogenic fungi have been shown to alter the behavior of the host insect, reducing locomotion, feeding and increasing irritability^54^. Grubs suffering from a fungal infection may not be able to feed or utilize food normally. This could adversely affect morphological, behavioral and physiological mechanism that grubs have evolved in order to defend against natural enemies in the soil^55^, thereby making the grub more susceptible to nematode penetration. Synergistic interactions between fungi and nematodes have been reported by many researchers^34–37, 56–58^. Contrarily^59^ found antagonism between EPNs and *Isaria fumosorosea* (Wize) when virulence to pecan weevil, *Curculio caryae* (Horn). Also, in contrast to this study^59^, also observed antagonism between certain combinations of *B. bassiana* and EPN, *Steinernema carpocapsae* (Weiser) or *Heterorhabditis indica* Poinar, Karunakar & David, and antagonism in combinations of *S. carpocapsae* and *M. anisopliae*. These discrepancies emphasize that synergistic interactions depend heavily on the host target as well as the pathogens that are being combined, and timing of applications (simultaneous versus sequential).

Growth and development are required for successful completion of the insect life-cycle and reproduction. Any delay may render the insect susceptible to biotic and abiotic factors (such as natural enemies or environmental regimes) that ultimately limit their growth and development. In this regard larval stages are vulnerable towards such phenomena^60^. In our study, treatments caused reduced food consumption, reduced longevity of the adult stage, and reduced weights in life-stages, which thus affected the insect’s fecundity and survival into the next generation.

The present study showed that *B. bassiana* and *M. anisopliae* isolates in integration with *H. bacteriophora* under laboratory conditions caused high mortality against larvae of the red palm weevil. The pathogens exerted detrimental effects on survival, growth and development of different developmental stages of *R. ferrugineus*. Hence, integrated application of *H. bacteriophor*a in sequential manners with *B. bassiana* and *M. anisopliae* might be effectively used for the successful control of red palm weevil. This approach (of combined applications) promises to provide superior efficacy than previous attempts to control the insect pest with singly applied treatments but further research is needed (e.g. under field conditions) for confirmation of the method’s success in date palm orchards.

## Materials and Methods

### RPW collection and rearing

A survey was conducted for collection of *R. ferrugineus* in date palm growing areas of west Punjab, Pakistan. Different developmental stages (larvae, pupae and adults) were collected from fallen and infested date palm trees with the permission of farmers (owners). All the stages collected were kept separately in plastic jars until brought to Microbial Control Laboratory, Department of Entomology, University of Agriculture, Faisalabad (UAF), Pakistan. Larvae were fed with sets of sugarcane (*Saccharum officinarum* L.; Poales: Poaceae) and pupation occurred within the stalks, while shredded sugarcane pieces were offered to adults for feeding and as a substrate for oviposition. After pupation, pupal cocoons were kept in separate plastic jars for adult emergence. After adult emergence beetles were shifted to the adult’s jar for feeding, mating and oviposition. The colony was developed in plastic boxes (30 × 60 × 60 cm) having a lid covered with mesh wire gauze (60 mesh size, 10 cm diameter) in the middle for aeration. The adult’s diet was changed every three days, and used sugarcane pieces were kept in separate jars (8 × 8 × 12 cm) for egg hatching. After egg hatching neonate larvae were allowed to feed for some time in the same set, after 3 days larvae were transferred to new sugarcane sets for feeding and pupation. Larvae were shifted to the new sugarcane sets after every week until pupation. The rearing conditions were maintained at 25 ± 2 °C, 65 ± 5% RH and a 12:12 (D: L) hour photoperiod.

### Entomopathogenic nematodes

Infective juveniles (IJs) of *H. bacteriophora* culture were obtained from Microbial Control Laboratory and used for the bioassays against 2^nd^, 4^th^ and 6^th^ instars of *R. ferrugineus*. *H. bacteriophora* was cultured in the final instar *Galleria mellonella* L. (Lepidoptera: Pyralidae) following the procedures of Kaya and Stock^61^.

### Entomopathogenic fungi

Two isolates of entomopathogenic fungi *B*. *bassiana* (WG-11) and *M*. *anisopliae* (WG-02) used in the study were obtained from the culture collection of the Microbial Control Laboratory; the strains were originally isolated from soils of vegetables and crop fields, respectively. Culturing of the fungi was accomplished by inoculating Petri plates containing Potato Dextrose Agar (PDA) media (BD, France)^62^. Spore concentration of 1 × 10^6^ spore ml^−1^ was determined with a Neubauer haemocytometer.

### Treatment with entomopathogenic fungi

The larvae (2^nd^, 4^th^ and 6^th^ instars) were directly immersed in 100 ml conidial suspension for 60 s individually and both treatments and the control were applied in an aqueous solution of 0.01% Tween-80 (Merck, KGaA, Darmstadt, Germany)^63^; control larvae were dipped into a suspension of 0.01% Tween-80 (without fungal spores). The treated and control larvae were then individually moved to 150 ml cylindrical plastic cups, each measuring 6 cm in height and 6 cm diameter. The top of the cups were covered with a fine mesh in order to prevent the insects from escaping. A piece of 2 × 2 cm^2^ artificial diet (Agar, brewer’s yeast, wheat germ, corn flour, ascorbic acid, benzoic acid, amino acid-vitamin mix, chloramphenicol and nipagin)^64^ was kept in the center of each cup. The cups were kept at 27 ± 2 °C, 65 ± 5% RH and a 12:12 (D:L) hour photoperiod in an incubator (Sanyo, Japan). Three replicates of 10 larvae were treated to the fungal suspensions or control. Each cup was opened daily and checked for mortality, and the old diet was replaced with fresh artificial diet until insect mortality or pupation was observed. After the insect reached the last instar stage, dry coir (coconut) was provided to the surviving larvae for pupation. The entire bioassay was repeated thrice.

### Treatment with H. bacteriophora

Nematode suspensions were prepared with concentrations of 100 IJs ml^−1^ in glass jars and 1 ml of suspension was poured into the cylindrical plastic cups lined with Whatman filter paper (described above). After pouring 30 minutes allowing the nematodes to distribute evenly on the filter paper, a small piece of artificial diet 2 × 2 cm^2^ was placed in the middle of the cups as a food source. Ten larvae for each treatment were used individually in each cup and each treatment was replicated three times, while control treatment received 1 ml of distilled water (without nematodes). The cups were maintained at above mentioned environmental conditions. The cups were opened daily to note mortality until insect mortality or pupation was observed. After the insect reached the last instar stage, dry coir was provided to the surviving larvae for pupation. The entire bioassay was repeated thrice.

### Treatment with entomopathogenic fungi and nematodes

The fungi (*B. bassiana* or *M. anisopliae*) were tested in combination with *H. bacteriophora*; combinations of the two fungi together (*B. bassiana* plus *M. anisopliae*) were not tested. In combined treatments fungi and nematodes were applied simultaneously or at different time intervals as follows:

- *B. bassiana* or *M. anisopliae* plus *H. bacteriophora* were applied simultaneously: larvae were immersed in fungal suspensions and transferred to the cylindrical plastic cups lined with moistened filter paper treated with *H. bacteriophora* IJs, and maintained at the conditions described above.
- Insects were first inoculated with *B. bassiana* or *M. anisopliae*, maintained at 27 ± 2 °C and 65 ± 5% RH for one week, then transferred to cylindrical plastic cups lined with moistened filter paper treated with *H. bacteriophora* IJs, and maintained at above mentioned conditions.
- Insects were first inoculated with *B. bassiana* and *M. anisopliae*, maintained at 27 ± 2 °C and 65 ± 5% RH for two weeks, transferred to cylindrical plastic cups lined with moistened filter paper treated with *H. bacteriophora* IJs, and maintained at above mentioned conditions.
- Control insects were immersed in aqueous solution with 0.01% Tween-80 and maintained in cylindrical plastic cups lined with moistened filter paper using conditions stated above.

Larval mortality was recorded after one, two and three weeks post application. For all treatments, artificial diet was offered to the larvae as food source. Larvae that failed to respond on slight prodding by a blunt needle were considered dead. After the insect reached the last instar stage, dry coir was provided to the surviving larvae for pupation. Percent pupation, adult emergence and egg eclosion were also recorded.

### Effects of entomopathogens on *R. ferrugineus* development

The effects of sub-lethal entomopathogen concentrations on development of RPW was assessed. Fourth instars were exposed to the sub-lethal dose of fungal entomopathogens (1 × 10^4^ conidia ml^−1^) and *H. bacteriophora* (50 IJs ml^−1^). The sub-lethal dosages were determined through preliminary experimentation (unpublished data). The larvae were fed on artificial diet and transferred to the treatment cups. Dry coir was provided to the each larva before pupation for cocoon formation. Adult insects, upon emergence, were offered shredded sugarcane pieces. Developmental parameters of each stage were recorded including larval duration, larval weight, pre-pupal duration, pre-pupal weight, pupal duration, pupal weight, adult longevity (male and female) and adult weight (male and female).

### Effects of entomopathogens on larval development

An additional assessment was made on the impact of sub-lethal concentrations on the development of last instar RPW; this experiment only involved *B. bassiana* and *H. bacteriophora* (because these pathogens were found to be the most compatible in terms of synergistic interactions, see Results section). Last instar RPW were exposed to sub-lethal doses of *B. bassiana* (1 × 10^4^ conidia ml^−1^) and *H. bacteriophora* (50 IJs ml^−1^). Before exposure in all the treatments larvae were weighed first and transferred to the rearing cups with artificial diet. Larvae continued to feed until they pupated; the insects were maintained under experimental conditions at 25 ± 2 °C, 65 ± 5% RH and a (12: 12) L: D hour photoperiod. Every day until the larvae pupated in the control (14 d), larvae were changed to a new clean cup and a new piece of artificial diet was offered. Frass produced during this period was separated from vials using a fine camel-hair brush, and weighed. Diet left unused in each vial was recovered, oven dried at 80 °C, and weighed. Prior to the assay, diet in fifteen vials was dried to obtain an estimate of the dry weight. Diet consumption of each larva was thus determined by subtracting the mass after feeding from before feeding estimate. Cumulative weight gain of larvae was also determined. Three replicates of ten insects were used for each treatment and same count of larvae fed on normal diet served as untreated check. The entire experiment was repeated thrice.

### Statistical analysis

The fungus-nematode interactions (synergistic, additive or antagonistic) were based on a comparison of observed versus expected values of insect mortality^59^. Expected mortality was calculated using formula *P*_*E*_ = *P*_*0*_ + (1 − *P*_*0*_) (*P*_*1*_) + (1 − *P*_*0*_) (1 − *P*_*1*_) (*P*_*2*_), where *P*_*E*_ is the expected mortality of the combination, *P*_*0*_ is the control mortality, *P*_*1*_ is the mortality from one pathogen treatment applied alone, and *P*_*2*_ is the mortality from the other pathogen applied alone. A *X* ^*2*^ test was applied to the observed and expected results: *X* ^*2*^ = (*L*_*0*_ − *L*_*E*_)^2^/*L*_*E*_ + (*D*_*0*_ − *D*_*E*_)^2^/*D*_*E*_, where *L*_*0*_ is the number of living larvae observed, *L*_*E*_ the number of living larvae expected, *D*_*0*_ the number of dead larvae observed, and *D*_*E*_ the number of dead larvae expected. Interactions were additive if *X* ^*2*^ < 3.84, antagonistic if *X* ^*2*^ > 3.84 and *P*_*C*_ < *P*_*E*_, and synergistic if *X* ^*2*^ > 3.84 and *P*_*C*_ > *P*_*E*_, where *P*_*C*_ is the observed mortality from the combination and *P*_*E*_ is the expected mortality from the combination. Data for pupation, adult emergence, egg eclosion and developmental parameters were subjected to one way analysis of variance (ANOVA) in Minitab^65^; means were separated using Tukey’s Kramer test (HSD)^66^ at a 5% significance level. To inspect the impact of microbial agents on the diet consumption, cumulative weight gain and frass production, data were analyzed by repeated measures (Proc Glimmix); based on residual plots data that were log transformed (back-transformed means are presented in the associated figures).

### Ethical approval

This article does not contain any studies with human participants performed by any of the authors.
